# Mitral valve analysis adding a virtual semi-transparent annulus plane for detection of prolapsing segments

**DOI:** 10.1186/s12947-015-0019-2

**Published:** 2015-05-20

**Authors:** Karl-Andreas Dumont, Jørn Skaarud Karlsen, Thomas Helle-Valle, Arnt Eltvedt Fiane, Runar Lundblad, Stig Urheim

**Affiliations:** Department of Cardiothoracic Surgery, Rikshospitalet, Oslo University Hospital, Post Office Box 4950, Nydalen, 0424 Oslo Norway; Kalkulo AS, Martin Linges vei 17, 1364 Fornebu, Norway; Department of Cardiology, Rikshospitalet, Oslo University Hospital, Post Box 4950, Nydalen, 0424 Oslo Norway; Department of Cardiothoracic Surgery and Faculty of Medicine in Oslo, Rikshospitalet, Oslo University Hospital, Post Office Box 4950, Nydalen, 0424 Oslo Norway; Department of Cardiology and Institute for Surgical Research, Rikshospitalet, Oslo University Hospital, Post Office Box 4950, Nydalen, 0424 Oslo Norway

**Keywords:** Echocardiography, Valvular heart disease, Mitral annulus, 3D echocardiography

## Abstract

**Background:**

We hypothesized that a novel three-dimensional virtual semi-transparent annulus plane (3D VSAP) presented on a holographic screen can be used to visualize the prolapsing tissue in degenerative mitral valve disease and furthermore, provide us with geometrical data of the mitral valve apparatus. Phantom and patient studies were designed to demonstrate the feasibility of creating a semi-automatic, semi-transparent mitral annulus plane visualized on a holographic display.

**Methods:**

Ten pipe cleaners mimicking the mitral annulus with different shapes and three types of annuloplasty rings served as phantoms. We obtained 3D transoesophageal examination of the phantoms in a special designed box filled with water. Recordings were converted to the holographic display and a 3D VSAP was created. The ratio of the major and minor axes as well as the non-planar angles were calculated and compared with direct measures of the phantoms. Forty patients with degenerative mitral valve disease were then analyzed with 3D transthoracic echocardiography (TTE) and a 3D VSAP was created on the holographic display. A total of 240 segments were analyzed by two independent observers, one echo expert (observer I), and the other novice with limited echo experience (observer II). The two observers created the 3D VSAP in each patient before suggesting the valve pathology.

**Results:**

The major/minor axes ratio and non-planar angles by 3D VSAP correlated with direct measurements by r = 0.65, p < 0.02 and r = 0.99, p < 0.0001, respectively. The sensitivity and specificity of the 3D VSAP method in patients was 81 and 97 %, respectively (observer I) and for observer II 77 and 96 %, respectively. The accuracy and precisions were 93.9 and 89.4 %, respectively (observer I), 92.3 and 85.1 % (observer II). Mitral valve analysis adding a 3D VSAP was feasible with high accuracy and precision, providing a quick and less subjective method for diagnosing mitral valve prolapse. This novel method may improve preoperative diagnostics and may relieve a better understanding of the pathophysiology of mitral valve disease. Thus, based on the specific findings in each patient, a tailored surgical repair can be planned and hopefully enhance long-term repair patency in the future.

## Background

Degenerative mitral valve disease is a common disorder affecting around 2 % of the population [1]. Valve repair is the optimal treatment and is associated with improved quality of life with less morbidity as well as better long-term survival as opposed to replacement [2–5]. Intraoperative 2D and real-time 3D transoesophageal echocardiography (TEE) are applied to guide the procedure and confirm a good result [6, 7]. Essentially, the surgical technique involves either resection or chordal replacement, or the combination of both [8–11] and a prosthetic annuloplasty ring or band [9, 11, 12]. Different surgical repair techniques and annuloplasty rings that either maintain or restore the saddle-shape of mitral annulus are available today [13]. Successful repair depends on correct pre-treatment diagnostics, the repair technique and finally post-repair intra-operative evaluation by TEE. Increased knowledge of the geometry and function of the mitral valve apparatus may reduce the number of repair failures. In recent years, there has been enormous progress in medical imaging technologies and in particular 3D ultrasound [14]. A novel 3D holographic display has been developed and enables 3D visualization of real-time 3D ultrasound data without the need for special glasses [15]. We have recently demonstrated the feasibility of analyzing mitral valve pathology and thereby a potential application of the screen for improved visualization of the mitral valve apparatus [16].

In this proof of concept study, a prototype tool for visualization of a 3D virtual semi-transparent mitral annulus plane (3D VSAP) has been developed on the already existing 3D holographic display thereby increasing the ability to visualize the prolapsing segments of the mitral valve. The rationale of adding a virtual annulus plane is that it may enhance diagnostic accuracy of mitral valve disease, especially prolapses involving anterior leaflet, and furthermore, identify residual prolapses perioperatively, thus avoiding repair failures. We tested the feasibility of creating a virtual mitral annulus plane in a phantom and patient study.

## Methods

### Phantom study

**Fig. 1:**
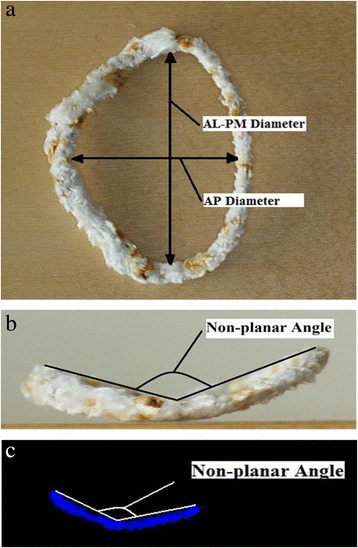
Pipe cleaner number 2 mimicking mitral annulus seen from front (**a**) and profile view (**b**). **c** Profile of the same pipe cleaner with 3D VSAP as presented on the holographic screen. *AL-PM* anterolateral-posteromedial. *AP* anteroposterior

Proof of concept was tested with use of pipe cleaners, mimicking the mitral annulus (Fig. 1a and b). We obtained TEE recordings (6 VT, GE Healthcare) in a special designed box filled with water from a total of 10 pipe cleaners with different shapes and non-planar angles and three different mitral annuloplasty rings: Edwards Lifesciences Corporation Physio® I (36 mm), Physio® II (34 mm) and Edwards IMR® ring (30 mm). Data sets were stored as (VolDicom) and exported to a separate workstation connected to a holographic screen (Setred AS) for further analysis. The files containing volumetric ultrasound data needed to be decompressed before being shown on the 3D display. The decompression was done using standard DICOM tool. The full volume dataset was presented in three dimensions (x, y, z) on the display [16].

**Fig. 2:**
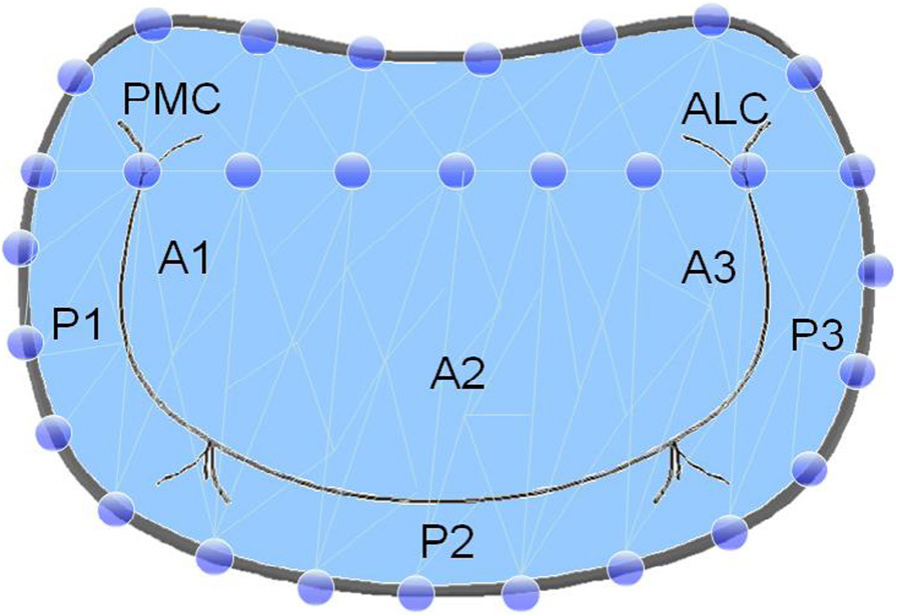
Shematic illustration of a phantom mimicking the mitral valve (Surgeon view) after labeling the circumference by mouse clicking (circular blue points) in a cropping plane perpendicular to the virtual plane. A line was drawn by clicking out points from “commissure to commissure“ (PMC to ALC) along the curvature of the phantoms to ensure the saddle shape of the annulus plane. A series of small triangles (Delaunay Triangulation) between the points generated the 3D surface that was made semitransparent (blue) to better appreciate the prolapsing tissue crossing the blue surface. *ALC:* Anterolateral commissure *PMC:* Posteromedial commissure

**Fig. 3:**
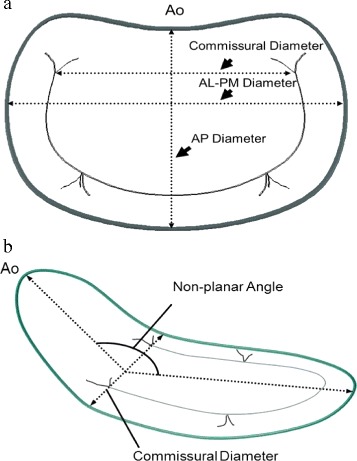
Mitral valve and its corresponding measurements front view (**a**) and profile (**b**). *AL-PM* anterolateral-posteromedial. *AP* anteroposterior. *Ao* aorta

The virtual plane on the holographic screen was derived by manually tracking the pipe cleaners along the “*anteroposterior”* cropping plane. This tracking consists of consecutively clicking out points along the circumference of the pipe cleaners, thus defining the external boundary of the virtual plane. Before tracking, a virtual line was drawn by clicking out points from “commissure to commissure “along the curvature of the phantoms to ensure the saddle shape of the annulus plane. The plane was finally made using a “*Delaunay triangulation process*” on all the points (Fig. 2) and was visualized as a three-dimensional semi-transparent blue surface. Typically, 20–25 points were needed to generate the 3D virtual plane. The signal intensity was adjusted leaving only the semi-transparent mitral annulus plane visible on the screen. Since we were not able to perform direct measurements on the 3D display, the annulus plane was projected into a 2D image showing the annulus plane in a front view (surgeon view) and side view (cropping plane perpendicular to the “anteroposterior” cropping plane) (Fig. 1c). The 2D images were stored in Paint (Microsoft®) and the ratio of the projected anterolateral-posteromedial and anteroposterior diameters (AL-PM/AP) as well as the non-planar angles for each pipe cleaner and annuloplasty ring were measured (Image J) (Fig. 3). Direct measurements of the phantoms (Image J) where obtained from photos of all pipe cleaners and annuloplasty rings (front and profile view similar to the projections from the 3D display).

### Patient study

#### Study population

Patients with mitral valve regurgitation were prospectively recruited from the outpatient cardiac clinic at Oslo University Hospital, Rikshospitalet. Criteria for enrollment were degenerative mitral valve disease including patients with Barlow disease scheduled for valve surgery. Patients with arrhythmia were excluded. All patients gave written informed consent. The study protocol was approved by the regional Committees on Biomedical Research Ethics. A total of 40 patients were included, of these 6 patients with Barlow disease.

#### Echocardiography

Echocardiographic examinations were obtained at the Department of Cardiology, Oslo University Hospital, Rikshospitalet. Two dimensional transthoracic and transoesophageal echocardiographic recordings were obtained by a Vivid E9 (GE Healthcare, Horten, Norway). Blinded to the 2D recordings, real time 3D echo acquisition of the mitral valve apparatus was obtained using a 3D volumetric transducer (4 V, GE Healthcare). A full-volume scan was acquired from an apical approach (frame rate of 27.2 ± 8.1 frames per second and number of heart cycles obtained 3.9 ± 0.76). The patient was tilted approximately 45 towards a left lateral decubitus position. Data sets were stored digitally in raw data format (VolDicom) and exported to the holographic screen.

The full volume dataset was presented in three dimensions (x,y,z) on the 3D display. In addition, a dynamic presentation was feasible, adding the fourth dimension (time) to the analysis. The cine loop covered a complete heart cycle and the loop was manually stopped at a time frame in systole when the prolapse was most prominent for further analysis. A similar approach as used for the phantom study was applied when generating the 3D virtual plane in patients. The cropping plane, however, was approximately perpendicular to the AP-diameter, and we tracked the mitral annulus by moving the cropping plane along the AL-PM axis. As for the phantoms, 20–25 points were used to generate the virtual plane. The description of valve pathology was in accordance with Carpentier’s classification [17].

A total of 240 mitral valve segments from 40 patients were analyzed by two independent observers. One observer was experienced echocardiographer (Observer I), the other novice with limited echocardiographic training (Observer II). Findings were compared with visual inspection during surgery in 35 patients and from 2D TEE in 5 patients.

### Statistics

Summary values are expressed as mean with standard deviation. Agreement between readers was measured using Cohen’s Kappa (Douglas G. Altman et al. Practical Statistics for Medical Research Chapman & Hall/CRC, 1991 pp 403–409). Grading of Kappa values was set at poor for 0–0.2; fair for 0.21–0.41; moderate for 0.41–0.6; good for 0.61–0.8 and very good for 0.81–1.0. Accuracy and precision was calculated according to the International vocabulary of metrology (JCGM 200:2008 International vocabulary of metrology - Basic and general concepts and associated terms (VIM)) according to the formula: Accuracy = (number of true positives + true negatives)/(number of true positives + true negatives + false negatives + true negatives) and precicion = number of true positives/(number of true positives + false positives). The AL-PM/AP ratio and non-planar angle measurements by the two methods (3D VSAP and direct measurements) were compared by use of the method of Bland and Altman [18] and by regression analysis with a least-squares method. We considered results significant at a value of p < 0.05.

## Results

### Phantom study

**Fig. 4:**
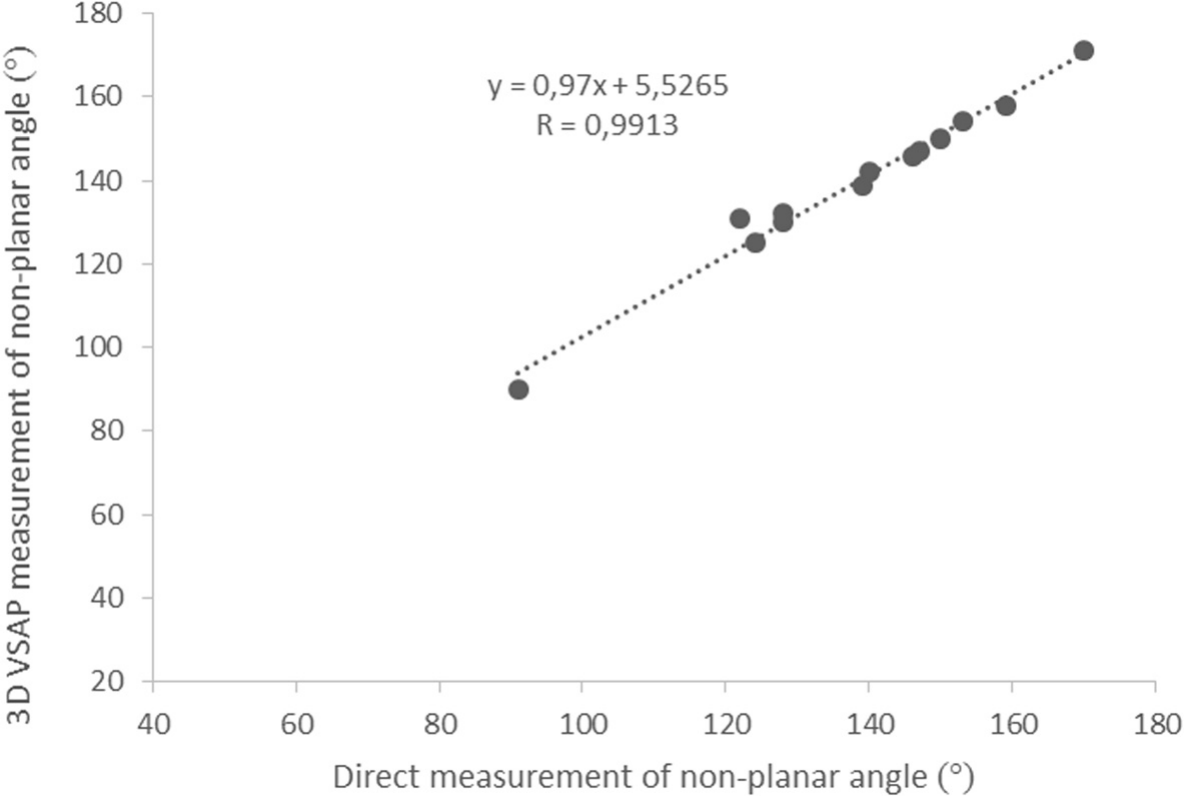
Correlation between non-planar angle in pipe cleaners and annuloplasty rings determined by 3D VSAP and direct measurements. y = 0.97x + 5.5265, r = 0.99, p < 0.0001

**Fig. 5:**
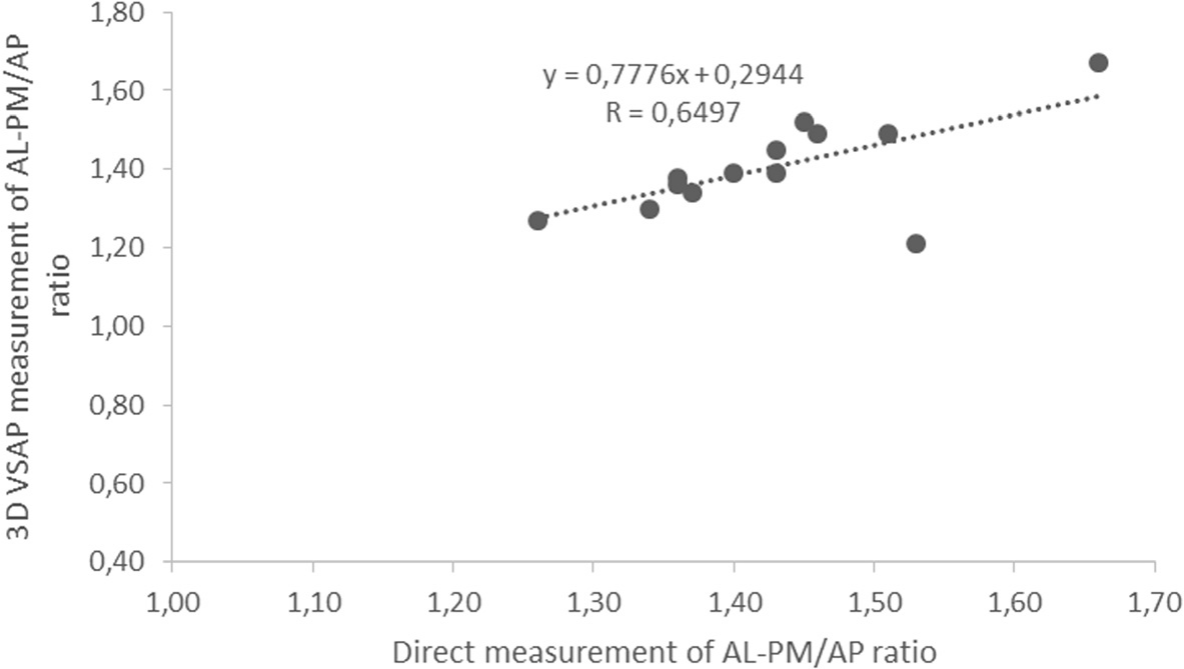
Correlation between AL-PM/AP ratio in pipe cleaners and annuloplasty rings determined by 3D VSAP and direct measurements. y = 0.7776x + 0.2944, r = 0.65, p < 0.02

**Fig. 6:**
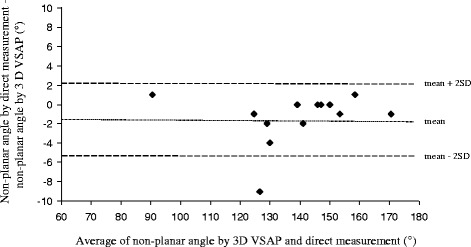
Agreement between non-planar angle by 3D VSAP and direct measurements. Mean difference between methods and ± 2 SD are indicated

**Fig. 7:**
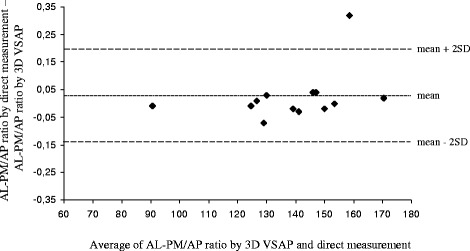
Agreement between AL-PM/AP ratio by 3D VSAP and direct measurements. Mean difference between methods and ± 2 SD are indicated

Table 1 shows the individual measurements of the pipe cleaners and annuloplasty rings (AL-PM/AP ratio and the non-planar angle) obtained by 3D VSAP method and direct measurements. The non-planar angles and AL-PM/AP ratios by 3D VSAP correlated with direct measurements by y = 0.97x + 5.5265, r = 0.99, p < 0.0001 and y = 0.7776x + 0.2944, r = 0.65, p < 0.02, respectively (Figs. 4 and 5). The Bland-Altman plots in Figs. 6 and 7 show the agreements between the non-planar angles and AL-PM/AP ratios, respectively.

**Table 1:** Ratio of major/minor axes (AL-PM/AP) and non-planar angles by 3D VSAP and direct measurements in pipe cleaners and the mitral annuloplasty rings

Pipe cleaner		Echo with 3D VSAP		Direct measurements	
Number		AL-PM/AP Ratio	Non-planar angle	AL-PM/AP Ratio	Non-planar angle
1		1.38	139	1.36	139
2		1.27	125	1.26	124
3		1.52	130	1.45	128
4		1.67	90	1.66	91
5		1.39	131	1.40	122
6		1.21	158	1.53	159
7		1.34	132	1.37	128
8		1.36	154	1.36	153
9		1.45	150	1.43	150
10		1.39	146	1.43	146
Annuloplasty rings		Echo with 3D VSAP		Direct measurements	
Type	Size	AL-PM/AP Ratio	Non-planar angle	AL-PM/AP Ratio	Non-planar angle
Edwards Physio® I	36 mm	1.49	142	1.46	140
Edwards Physio® II	34 mm	1.30	147	1.34	147
Edwards IMR® ring	30 mm	1.49	171	1.51	170

*AL-PM* anterolateral-posteromedial diameter, *AP* anteroposterior diameter, *VSAP* virtual semi-transparent annulus plane

### Patient study

Table 2 shows the basic echocardiographic findings in all patients. We were able to obtain an annulus plane in all subjects. Table 3 summarizes the results from mitral valve segment analysis by visual inspection during surgery and with 3D VSAP method by both observers. A

**Table 2:** Haemodynamic parameters

Age (years)	57 ± 15
Male (%)	72
Heart rate (bpm)	70 ± 12
LVIDd (mm)	63.3 ± 6.9
LVIDd indexed (mm/m^2^)	33.4 ± 3.8
LVIDs (mm)	40.3 ± 5.4
LVIDs indexed (mm/m^2^)	21.3 ± 3.2
LV EDV (ml)	211.3 ± 48.0
LV EDV indexed (ml/m^2^)	111.1 ± 22.5
LV ESV (ml)	80.4 ± 25.3
LV ESV indexed (ml/m^2^)	42.5 ± 12.2
LV Stroke volume (ml)	68.7 ± 16.9
LV Cardiac output (l/min)	4.7 ± 0.94
LV EF (%)	62 ± 4.8
LA area (mm^2^)	27.7 ± 6.4
MAD (AP) (mm)	3.95 ± 0.4
Regurgitant volume (ml/beat)	62.2 ± 26.7

*Bpm*, beats per minute, *LVIDd*, left ventricular internal dimension diastole, *LVIDs*, left ventricular internal dimension systole, *LV SV*, left ventricular stroke volume, *LV EF*, left ventricular ejection fraction, *LV EDV*, left ventricular end-diastolic volume, *LV ESV*, left ventricular end-systolic volume, *LA*, left atrium, *MAD (AP)*, mitral annulus diameter (antero-posterior)

**Table 3:** Prolapsing segments suggested by the two observers (number of correct segments in parenthesis)

Prolapsing segments	Surgical findings	Observer I	Observer II
A1	1	0	0
A2	8	8(7)	5(5)
A3	6	5(4)	7(4)
P1	2	1(0)	1(0)
P2	31	31(30)	30(29)
P3	5	2(2)	5(2)
Number	53	47	48
Sensitivity		0.81	0.77
Specificity		0.97	0.96

total of 240 segments were analyzed and visual inspection during surgery revealed a total of 16 prolapsing segments. Both observers spent 2–6 min to complete the virtual annulus plane and describe the valve pathology. Figures 8a and 9a show representative recordings of different mitral valve pathology (surgeon view) and demonstrates where the prolapsing segments crosses the visualized plane. The sensitivity and specificity of the 3D VSAP method was 81 and 97 %, respectively (observer I) and for observer II 77 and 96 %, respectively. The accuracy and precisions were 93.9 and 89.4 %, respectively (observer I), 92.3 and 85.1 % (observer II). The inter-observer agreement was 0.96 with Cohen’s Kappa 0.86.

**Fig. 8:**
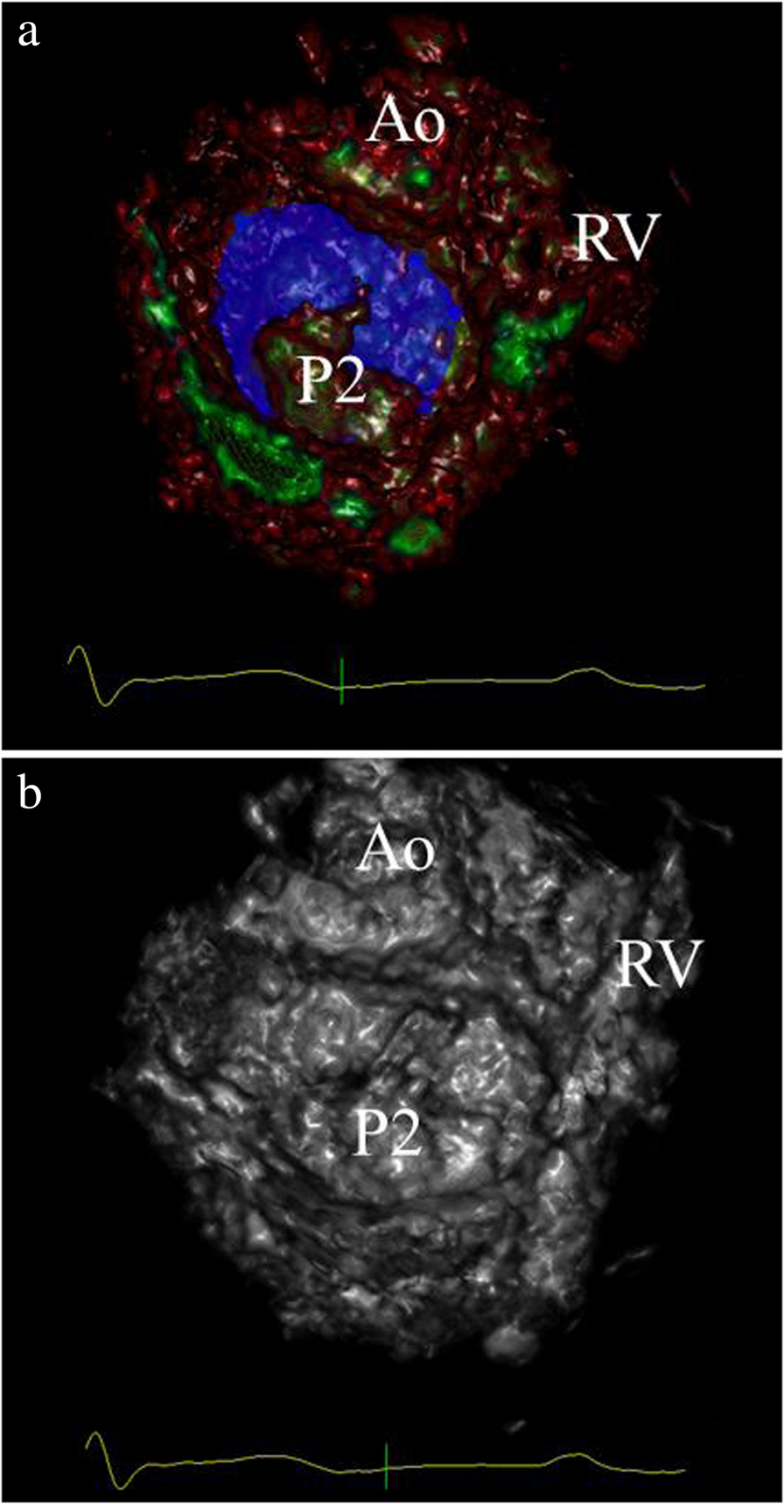
Surgeon view of the mitral valve from a patient with P2 prolapse as presented on the holographic screen. 3D VSAP is visible as a semi-transparent blue plane showing clearly the prolapsing segment that crosses the surface (**a**). **b** The same patient without 3D VSAP. *Ao* aorta, *RV* right ventricle, *P2* indicating prolapse of mid segment posterior leaflet

**Fig. 9:**
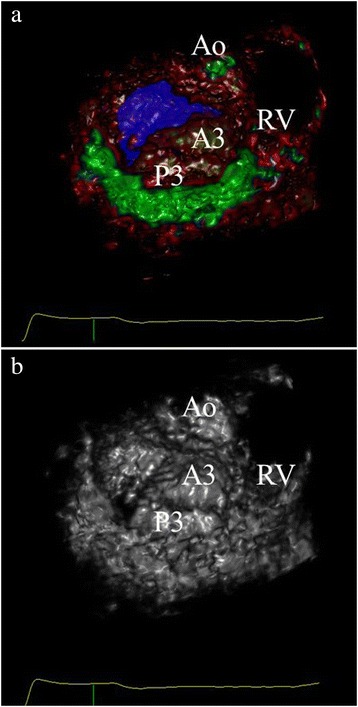
Surgeon view of the mitral valve from a patient with A3/P3 prolapse as presented on the holographic screen. 3D VSAP is visible as a semi-transparent blue plane showing clearly the prolapsing segments that crosses the surface (**a**). **b** The same patient without 3D VSAP. *Ao* aorta, *RV* right ventricle, A3 and P3 indicating prolapse of the posteromedial segments of the mitral leaflets

## Discussion

The introduction of 3D echocardiography opens for improved diagnostics and treatment of valvular heart disease. Still, the exact extension of prolapsing tissue in degenerative mitral valve disease may be challenging, particularly in Barlow’s disease, and the use of a virtual annulus plane may be beneficial. Perioperative 3D VSAP may improve the diagnostic accuracy of on-table quality control after mitral valve repair.

The present study demonstrates proof of concept and shows good agreement between the 3D VSAP presented on the holographic screen and the configuration of the phantoms (pipe cleaners and annuloplasty rings). Furthermore, we showed in patients that the semitransparent annulus plane could easily visualize which part of the leaflets that was crossing the plane (Figs. 8 and 9) in good agreement with the findings during surgery. More important, a very good inter-observer agreement was demonstrated, indicating that a novice with limited echocardiographic experience can perform advanced segment analysis similar to the expert.

The importance of analyzing the annulus plane in mitral regurgitation has been addressed in several studies the recent years [19–24]. Both animal studies and simulations have shown the effect of different annuloplasty rings on mitral leaflet dynamic motion [24] and leaflet strains [20]. The present 3D VSAP method may enable better and rapidly mitral annular assessment, ensuring appropriate annuloplasty selection and perioperative assessment of the valve repair We demonstrated the feasibility of creating an “annulus plane” in phantoms (pipe cleaners and annuloplasty rings) and the measured non-planar angles and dimensions (ratios) correlated well with direct measurements. This could open for a more robust estimation of the annulus plane after implantation of the ring perioperatively, and may expose small residual prolapses that need to be addressed on-table.

When proven robust, the method can be applied in multicenter studies to demonstrate whether use of a virtual annulus plane provides better short- and long-term repair patency.

The patient study was obtained with 3D TTE. The reason for using TTE recordings, was that 3D TEE files were too large to be transferred to the holographic screen. This will be solved in the near future and the segmentation of the mitral annulus is expected to be easier with better acoustic windows. Furthermore, the virtual plane is only visible in one time frame, therefore tracking algorithms will be developed to show the plane moving throughout the heart cycle providing dynamic information about the deformity and rigidity of the mitral annulus.

## Conclusion

The presentation of a semi-transparent annulus plane was feasible, and the estimated non-planar angle in phantoms correlated well with direct measurements. In patients, we were able to obtain the virtual mitral plane in all, visualizing the prolapsing segments with a good accuracy and precision by both observers. A potential application of this method is more precise localization and quantification of prolapsing leaflet tissue in mitral valve disease.

## References

[1] Enriquez-Sarano M, Akins CW, Vahanian A (2009). Mitral regurgitation. Lancet.

[2] David TE, Ivanov J, Armstrong S, Rakowski H (2003). Late outcomes of mitral valve repair for floppy valves: implications for asymptomatic patients. J Thorac Cardiovasc Surg.

[3] Enriquez-Sarano M, Schaff HV, Orszulak TA, Tajik AJ, Bailey KR, Frye RL (1995). Valve repair improves the outcome of surgery for mitral regurgitation. A multivariate analysis. Circulation.

[4] Gillinov AM, Blackstone EH, Nowicki ER, Slisatkorn W, Al-Dossari G, Johnston DR (2008). Valve repair versus valve replacement for degenerative mitral valve disease. J Thorac Cardiovasc Surg.

[5] Suri RM, Schaff HV, Dearani JA, Sundt TM, Daly RC, Mullany JC (2006). Survival advantage and improved durability of mitral repair for leaflet prolapse subsets in the current era. Ann Thorac Surg.

[6] Adams DH, Anyanwu AC, Sugeng L, Lang RM (2008). Degenerative mitral valve regurgitation: surgical echocardiography. Curr Cardiol Rep.

[7] O’Gara P, Sugeng L, Lang R, Sarano M, Hung J, Raman S (2008). The role of imaging in chronic degenerative mitral regurgitation. JACC Cardiovasc Imaging.

[8] Braunberger E, Deloche A, Berrebi A, Abdallah F, Celestin JA, Meimoun P (2001). Very long-term results (more than 20 years) of valve repair with carpentier’s techniques in nonrheumatic mitral valve insufficiency. Circulation.

[9] Carpentier A, Deloche A, Dauptain J, Soyer R, Blondeau P, Piwnica A (1971). A new reconstructive operation for correction of mitral and tricuspid insufficiency. J Thorac Cardiovasc Surg.

[10] Filsoufi F, Carpentier A (2007). Principles of reconstructive surgery in degenerative mitral valve disease. Semin Thorac Cardiovasc Surg.

[11] Carpentier AF, Lessana A, Relland JY, Belli E, Mihaileanu S, Berrebi AJ (1995). The “physio-ring”: an advanced concept in mitral valve annuloplasty. Ann Thorac Surg.

[12] Gillinov AM, Cosgrove DM, Shiota T, Qin J, Tsujino H, Stewart WJ (2000). Cosgrove-edwards annuloplasty system: midterm results. Ann Thorac Surg.

[13] Salgo IS, Gorman JH, Gorman RC, Jackson BM, Bowen FW, Plappert T (2002). Effect of annular shape on leaflet curvature in reducing mitral leaflet stress. Circulation.

[14] Urheim S, Andersen K, Aakhus S (2012). Three-dimensional ultrasound in cardiological diagnostics. Tidsskr Nor Laegeforen.

[15] Abildgaard A, Witwit AK, Karlsen JS, Jacobsen EA, Tennøe B, Ringstad G (2010). An autostereoscopic 3D display can improve visualization of 3D models from intracranial MR angiography. Int J Comput Assist Radiol Surg.

[16] Beitnes JO, Klæboe LG, Skaarud Karlsen J, Urheim S (2015). Mitral valve analysis using a novel 3D holographic display – a feasibility study of 3D ultrasound data converted to a holographic screen. Int J Cardiovasc Imaging.

[17] Carpentier A (1983). Cardiac valve surgery–the “French correction”. J Thorac Cardiovasc Surg.

[18] Bland JM B, Altman DG (1986). Statistical methods for assessing agreement between two methods of clinical measurement. Lancet.

[19] Blanke P, Dvir D, Cheung A, Ye J, Levine RA, Precious B (2014). A simplified D-shaped model of the mitral annulus to facilitate CT-based sizing before transcatheter mitral valve implantation. J Cardiovasc Comput Tomogr.

[20] Bothe W, Kuhl E, Kvitting JP, Rausch MK, Göktepe S, Swanson JC (2011). Rigid, complete annuloplasty rings increase anterior mitral leaflet strains in the normal beating ovine heart. Circulation.

[21] Bothe W, Rausch MK, Kvitting JP, Echtner DK, Walther M, Ingels NB (2012). How do annuloplasty rings affect mitral annular strains in the normal beating ovine heart?. Circulation.

[22] Lin QS, Fang F, Yu CM, Zhang YC, Hsiung MC, Salgo IS (2014). Dynamic assessment of the changing geometry of the mitral apparatus in 3D could stratify abnormalities in functional mitral regurgitation and potentially guide therapy. Int J Cardiol.

[23] Rausch MK, Bothe W, Kvitting JP, Swanson JC, Ingels NB, Miller DC (2011). Characterization of mitral valve annular dynamics in the beating heart. Ann Biomed Eng.

[24] Bothe W, Kvitting JP, Swanson JC, Göktepe S, Vo KN, Ingels NB (2010). How do annuloplasty rings affect mitral leaflet dynamic motion?. Eur J Cardiothorac Surg.

